# Microbial Primer: The logic of bacterial plasmids

**DOI:** 10.1099/mic.0.001336

**Published:** 2023-07-03

**Authors:** Georgina S. Lloyd, Christopher M. Thomas

**Affiliations:** ^1^​ School of Biosciences, University of Birmingham, Edgbaston, Birmingham B15 2TT, UK

**Keywords:** plasmid, mobile genetic element, horizontal gene transfer, autonomous replication, conjugative transfer, extrachromosomal element

## Abstract

This short primer is intended to give an overview of bacterial plasmids for those not yet familiar with these fascinating genetic elements. It covers their basic properties but does not attempt to cover the diversity of phenotypic properties that can be encoded by plasmids, and includes suggestions for further reading.

## Definition

Plasmids are non-essential genetic elements that can replicate without integrating into the chromosome of their host. They are often described as replicating independently or autonomously but no known plasmids encode the complete machinery needed for replication – they generally depend on host enzymes, for example DNA polymerase or DNA gyrase. They can range in size from as little as 1 kb to hundreds of kilobase pairs, which is as big as the smallest chromosomes. They became the workhorses of modern biotechnology after underpinning the invention of recombinant DNA technology and gene cloning in the 1970s.

**Fig. 1.**
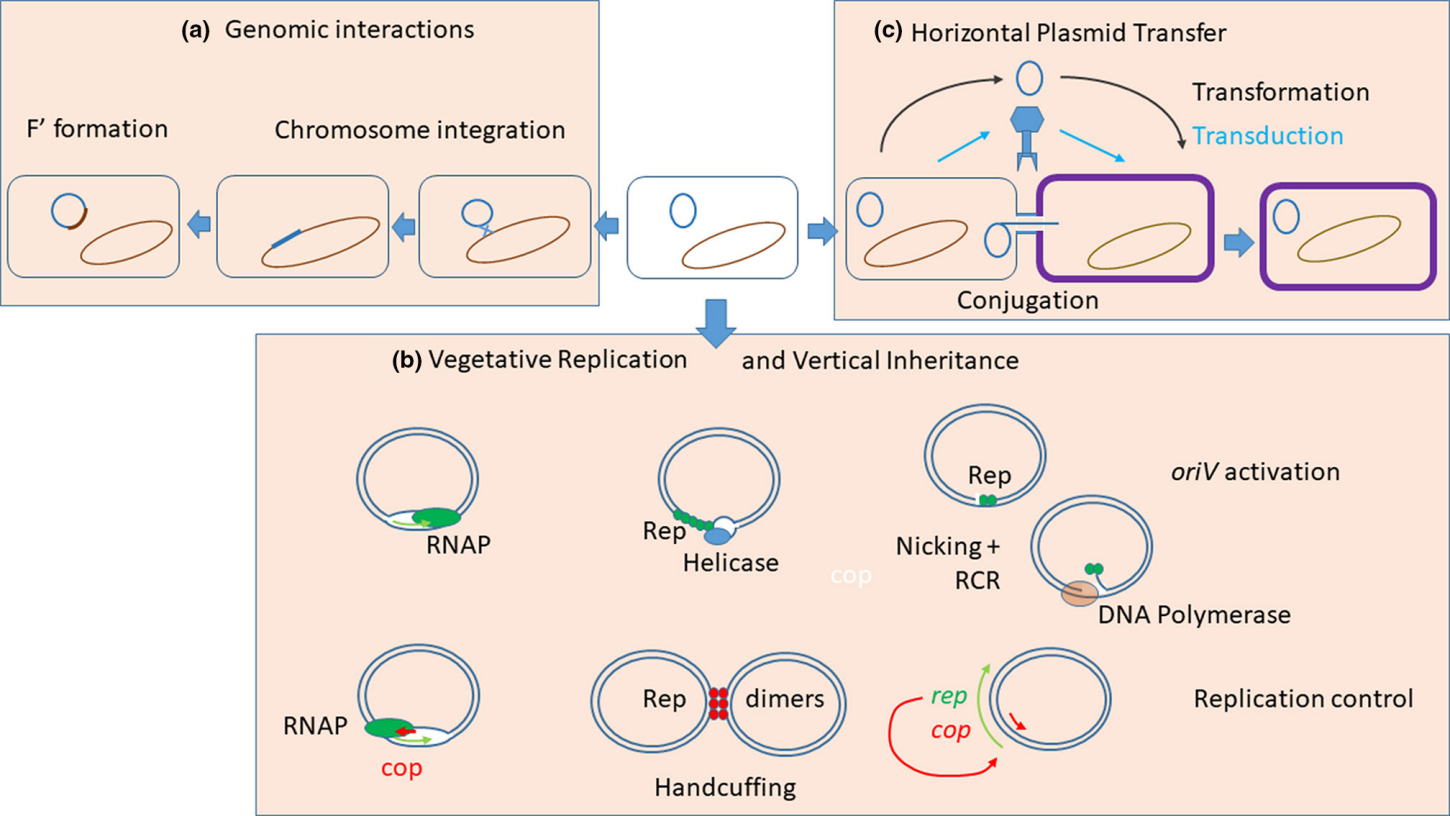
Summary of key plasmid properties including interaction with the chromosome (**a**), vertical inheritance via vegetative replication (**b**) and horizontal transfer to different bacteria (**c**). As much as possible is explained by labelling on the Figure. Recombination between plasmid and chromosome to allow integration normally occurs where the plasmid and chromosome have identical transposable elements. Rep protein monomers and dimers are shown in green where they activate *oriV*, leading in helicase to unwind *oriV* or nick *oriV* to initiate rolling circle replication (RCR). Rep dimers are shown in red where their role is negative – by ‘handcuffing’. The Rep protein can also sometimes autoregulate as shown by the red arrow; *cop* (shown in red) indicates copy number control genes producing antisense RNA.

As a result of recombination or transposition, plasmids can gain genes from the chromosome, and this can also result in loss of genes from the chromosome as for example occurs with the F plasmid (the original Fertility factor that was discovered to promote gene exchange between *Escherichia coli* strains) when an F′ plasmid is generated (Fig. 1a). However, in this situation, if the acquired segment is essential for cellular function, then the plasmid becomes essential and thus no longer fits the definition of a plasmid. The term ‘chromid’ is used for an element that has core sequences like known plasmids but carries essential functions. An example is chromosome 2 in *Vibrio cholerae *.

Although there are many plasmid lineages, the set of genetic and biochemical functions that plasmids encode to be successful and how these are organized follow a logic that is observed repeatedly. With the increasing development of synthetic and semi-synthetic plasmids as vehicles to carry functional gene cassettes into complex microbiomes, that logic needs to be understood and respected to avoid unnecessary pitfalls. This primer summarizes the logic that has been uncovered over the last 50 years concerning essential and beneficial plasmid backbone functions, as well as their organization in the plasmid genome, to provide a guide for those wanting to understand or engineer plasmids.

## Replication

Plasmids are normally double stranded circular or linear molecules. For linear plasmids, the ends can be protected by a covalently closed hairpin loop or covalently attached protein. Replication is the essential property that all plasmids must possess, and the plasmid region(s) needed for replication is called the ‘replicon’. Much research has focused on understanding how plasmids replicate in a controlled way so that the average number of plasmid copies doubles each host generation. Like bacterial chromosomes, plasmids normally have just one specific place (or occasionally two or three) where replication is initiated called the origin (*oriV*, with V for vegetative or vertical – non-sexual – reproduction) and they control that process by a negative feedback loop.

Replication is normally achieved in one of three ways (Fig. 1b). First, by transcribing the *oriV* region to give an RNA molecule that is processed by ribonucleases to form the primer for leading strand synthesis at *oriV*. Second, by producing a protein (Rep) that binds to multiple sites (called iterons) in *oriV* and promotes strand separation followed by primer formation to initiate replication. Generally, leading strand initiation is followed by primosome assembly on the lagging strand to create a standard replication fork at which both strands are copied. However, some plasmids lack primosome assembly sites and rely on displacement of the lagging strand, which remains single stranded (ssDNA) until the divergent initiation site at *oriV* allows synthesis of the complementary strand. The best examples of this strategy are IncQ plasmids that are about 10 kb in size. When such plasmids have been used as vectors for large DNA segments, they are unstable due to the detrimental effects of ssDNA accumulation. The logical solution to this if one wants to exploit the replication system because of its broad host range would be to incorporate a primosome assembly site on the lagging strand close to *oriV*. The third initiation strategy is to produce a Rep protein that cuts one strand at *oriV* to create a 3′ end to initiate rolling circle replication (RCR). Replication can be either unidirectional, starting and ending at *oriV*, or bidirectional from *oriV* to a terminus.

Control of replication is provided by mechanisms that decrease the probability of replication as the copy number of the plasmid increases (Fig. 1b). Often this is achieved by the plasmid producing constitutively an anti-sense RNA and/or protein repressor that interferes with *oriV* activation, either by preventing transcript processing or by blocking Rep protein production. Additionally, the Rep protein may activate *oriV* as a monomer but repress it as a dimer by linking *oriV*s (‘handcuffing’) or creating intramolecular loops that block activation.

## Stable inheritance

Inserting a conditionally lethal gene (such as *sacB* encoding the enzyme levan sucrase which polymerizes sucrose in the periplasm of Gram-negative bacteria, blocking cell wall biosynthesis) allows one to detect even low plasmid loss rates per generation. Successful plasmids have acquired genes that minimize this loss. Small plasmids tend to be found at medium (15) or high (>30) copy number per cell and rely mainly on random segregation for stability. However, homologous recombination between plasmid copies, sometimes stimulated by DNA damage, creates multimers consisting of two or more directly repeated plasmid genomes. This reduces the number of separate plasmid molecules and increases the random plasmid loss rate. Therefore, plasmids commonly encode a multimer resolution system (MRS) promoting site-specific recombination when two sites encounter each other in a supercoiled, circular multimer. Interestingly, plasmids that use an RCR mechanism generally do not carry an MRS because the RCR process generates monomers, even from a multimer template.

Many plasmids have also acquired addiction systems encoding both a stable toxin and a related resistance mechanism which may be unstable. For example, the widespread *parDE* genes consist of *parE* encoding a stable toxin that inhibits DNA gyrase while *parD* encodes an unstable autoregulatory repressor and antitoxin protein that ensures ParE only works when the plasmid is lost, preventing survival of plasmid-free segregants. Other gene systems can also generate ‘addiction’; for example, many plasmids encode lethal bacteriocins plus an immunity protein. A few cells in a population produce bacteriocin in a self-lethal burst of production, killing any other bacteria not producing the plasmid-encoded immunity protein.

Most large plasmids have a low (one or two) copy number and would be lost rapidly if transmission was a random process. They therefore encode active partitioning (mitotic) machinery in one, or sometimes two, gene set(s), mostly commonly *parABS* or *parMRC*. For *parABS* systems, ParB binds to the centromere-like *parS* sequence and to ParA-ATP which binds chromosomal DNA in the nucleoid. This complex promotes ATP hydrolysis, weakening linkages to chromosomal DNA and ParB, creating a wave that moves the plasmid away from the centre of the cell. For *parMRC* systems, ParR binds to the centromere-like *parC* site and to ParM-ATP that polymerizes, creating a spindle that pushes the plasmids apart.

## Plasmid incompatibility and classification

As plasmids evolve, they may become associated with distinguishable phenotypes but retain essentially the same replication, copy number control and stable inheritance mechanism(s). If two such plasmids end up in the same cell they can form a common pool of replicating molecules that are randomly segregated when the host divides. The result is an imbalance in the numbers of the two plasmids and further generations will accentuate this so that eventually cells with just one or other plasmid are produced. This competition between related plasmids is called incompatibility and allowed researchers to classify plasmids into ‘incompatibility (or Inc) groups’. Such groups found in *E. coli* and other *Enterobacteriaceae* are named alphabetically (IncA, IncB, etc.) and other bacterial groups have developed similar but distinct systems. As plasmid DNA sequences became available it was recognized that plasmids in the same group had the same or similar replication or stable inheritance functions, so classification could be done by sequence similarity. With the growing wealth of DNA sequence information, it is now possible to discover and group plasmids about which we know very little. So how we classify plasmids is once again a major subject of debate.

## Horizontal transfer

Gene spread between clonal lineages is called horizontal gene transfer. Plasmids can transfer between bacteria: by transformation of naked DNA; by transduction when packaged into a bacteriophage particle; by budding of membranous vesicles; or by a process called conjugative transfer involving cell–cell contact and ‘mating bridge’ formation (Fig. 1c). Some plasmids have fused with a lysogenic bacteriophage, so they exist as a plasmid while a prophage but multiply as a virus when activated.

The best studied conjugative plasmids produce a protein tube called a pilus on the bacterial surface by a type IV secretion system (T4SS) essential for ‘mating bridge’ formation. A protein–plasmid DNA complex at the transfer origin (*oriT*) recognizes the bridge and nicks *oriT* to initiate RCR. This drives single-stranded plasmid DNA movement from donor to recipient and re-circularization once *oriT* is reached again. The complex at *oriT* is called a relaxosome because treatments that denature the nicking enzyme (relaxase) nick the DNA and relax the supercoils. Complementary strand synthesis is often initiated by a plasmid-encoded primase that transfers with the ssDNA from donor to recipient. Many genes are needed for this process and can occupy more than 40 kb. Some plasmids even use almost twice this genetic space because they encode multiple pili including a long flexible pilus that stabilizes mating pairs in liquid medium. These sets of transfer genes can also be classified, and it is interesting that some plasmids with basically the same transfer system can be associated with different replication systems. For some plasmids conjugative transfer is less sophisticated – some plasmids found in *Streptomyces* bacteria encode a single protein that promotes inter-hyphal transfer.

Not all plasmids are self-transmissible. Some small plasmids lack any conjugative functions while others, called ‘mobilizable’, can only transfer when suitable self-transmissible plasmids are present. Such plasmids carry only enough genetic information to create an active relaxosome from their *oriT* region and the Mob proteins they encode, allowing it to dock with and use the mating bridge formed by its co-resident plasmid.

## Anti-restriction, anti-CRISPR and other post-transfer functions

Restriction-modification and CRISPR systems can pose a barrier for the spread of plasmid DNA between bacteria – even within the same species. Unsurprisingly, many conjugative plasmids have acquired or evolved genes or other strategies that protect them from these threats. Three plasmid-encoded anti-restriction determinants (proteins) have been characterized in some detail and appear to work by binding to and blocking Type I restriction enzyme function. There is also clear evidence that evolutionary selection has resulted in many plasmids having lost many of the sites that make them susceptible to Type II restriction enzyme cleavage. Many anti-CRISPR functions have been identified in bacteriophages and similar functions are increasingly being found in plasmids as well. In addition, the leading region (that enters the recipient first during transfer) may carry genes that facilitate the process, for example by blocking induction of the SOS response triggered by ssDNA which is normally a sign that damaged DNA is being repaired. The orientation of these genes can allow them to be expressed from the single stranded plasmid DNA immediately after transfer before it has been converted to double stranded DNA.

## Phenotypic cargo

Plasmids that simply consist of genes that promote their own replication, stable inheritance and transfer are called ‘cryptic’ or ‘selfish’ because they do not provide an advantage for their hosts other than encouraging gene exchange. It is often assumed that all plasmids must be a burden to their hosts and that their existence is a paradox, but evolution experiments show that the cost of a plasmid can be minimized or ameliorated over time by genetic changes that modulate the host–plasmid relationship. However, this adaptation rarely seems to be for all hosts at the same time. So, many abundant plasmids have acquired genes such as antibiotic resistance genes that are not universal in bacteria and which promote the differential survival of their host. Plasmids gain from being the agents that spread such genes but it appears that long-term survival of these genes in their new hosts is best assured by integration into the chromosome. The simplest examples of plasmid-borne phenotypes are where a single gene confers a strongly advantageous phenotype, such as a beta-lactamase which gives penicillin resistance. More complex phenotypes such as catabolism of complex organic molecules or synthesis of antibiotics are also found on plasmids and can be rationalized as only being advantageous in localized habitats. Although there are some genes that are thought of as typically ‘plasmid-borne’, in practice almost anything can be found on a plasmid if you look hard enough.

## Organization

**Fig. 2.**
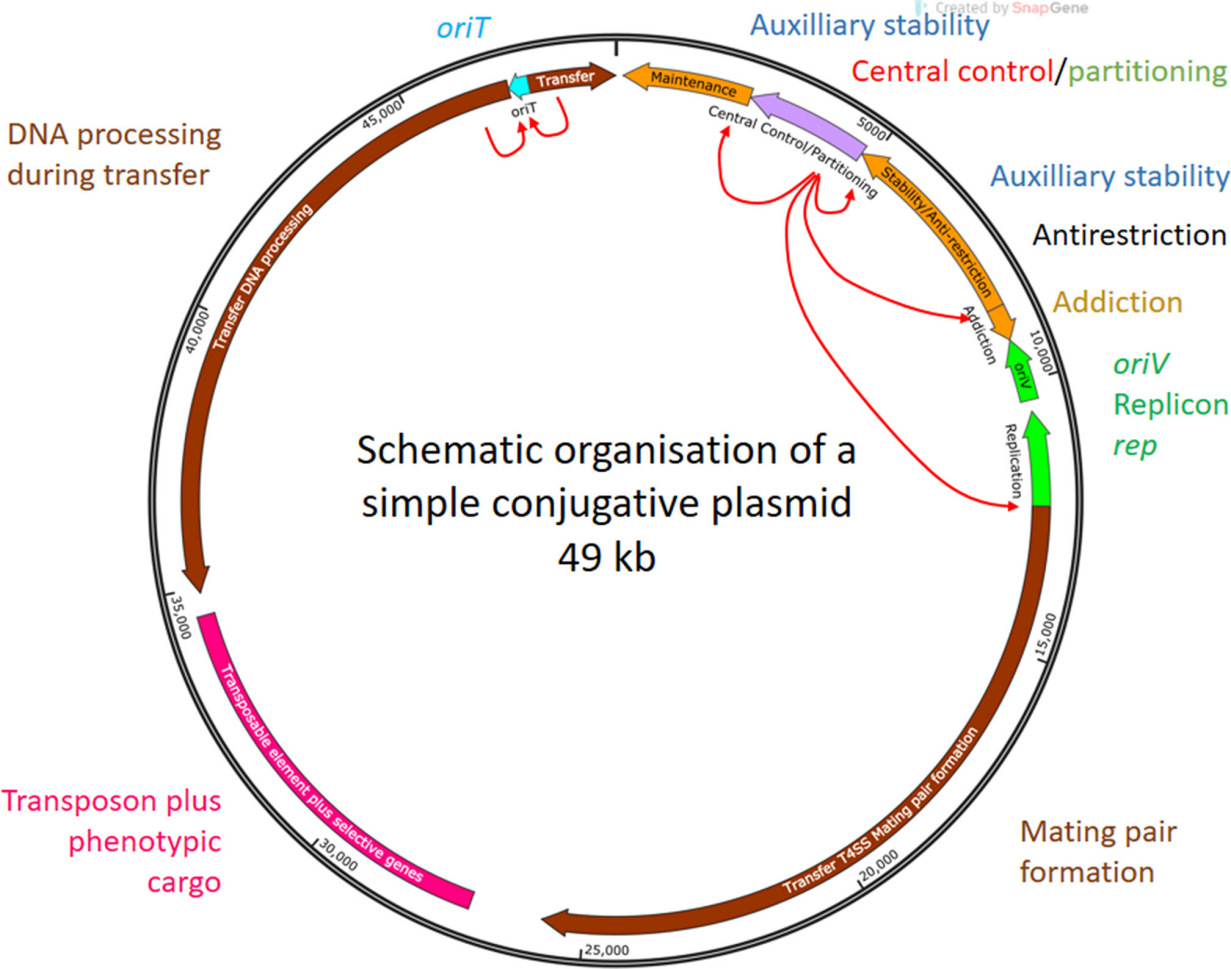
Distribution of the basic plasmid functions for a smallish conjugative plasmid. Arrows indicate the direction of vegetative (replication fork moves anticlockwise) and transfer replication (the region behind the arrow enters first, so RCR moves clockwise). This means that the leading region during transfer can potentially be transcribed and translated before transfer is complete and DNA is double stranded once again. The red arrows show regulatory circuits. A single block of phenotypic cargo is shown but many plasmids carry more than one. Such a plasmid would typically encode about 50 genes – on average about one per kilobase. The blocks shown represent transcriptional units (or a genetic element in the case of the transposable element) rather than individual genes.

Many plasmids show evidence of a set of basic replication, stable inheritance and transfer genes that came together in a functional way before being interrupted by acquisition of genes advantageous to their host. The IncP plasmids of Gram-negative bacteria are a good example of this, having all of the transcriptional units of this backbone regulated by a central control region which doubles up as the core of the partitioning apparatus as well (Fig. 2). It also illustrates how genes for the two modes of inheritance – vertical and horizontal – can be coordinated so that, on entry into a new cell, vegetative replication has priority but gives way to transfer when establishment is assured. A similar juxtaposition is observed in many other groups. Interruption of these backbones is only successful if insertion sites do not disrupt the core functions. Interestingly, in a subset of the IncP plasmids the gaps between functional blocks are marked by repeated sequences that appear to attract insertions and when a region has been interrupted once, it is often targeted again. One can also see logic in the orientation of core functions relative to each other. For example, the orientation of the genes transferred early during conjugative transfer generally allows them to be transcribed to yield functional mRNA before the completely double stranded plasmid is restored. Discovering the logic of such systems is richly rewarding both as an intellectual exercise and as a means of using the full functionality of these biotechnology workhorses.

## Further reading

1. Addgene (2017) *Plasmids 101*, Third Edition. www.addgene.org

2. Fernandez-Lopez R, Redondo S, Garcillan-Barcia MP, de la Cruz F (2017) Towards a taxonomy of conjugative plasmids. *Current Opinion in Microbiology* 38, 106–113.

3. Funnell BE, Phillips GJ (eds) (2004) *Plasmid Biology*. ASM Press. ISBN 1-55581-265-1

4. Hall JPJ, Harrison E, Baltrus D (eds) (2022) The secret lives of microbial mobile genetic elements. *Philosophical Transactions of the Royal Society B Biological Sciences* 377, Issue 1842.

5. Mace K, Vadakkepat, AK, Redzej A, Lukoyanova N, Oomen C, Braun N, Ukleja M, Lu F, Costa TRD, Orlova EV, Baker D, Cong, Q, Waksman G (2022) Cryo-EM structure of a type IV secretion system. *Nature* 607, 191–196.

6. Summers DK (2009) *The Biology of Plasmids*. John Wiley and Sons. ISBN 1444313738, 9781444313734.

7. Thomas CM (2000) *The Horizontal Gene Pool: Bacterial Plasmids and Gene Spread*. CRC Press. https://doi.org/10.1201/9781482283549

8. Thomas CM, Summers D (2020) Bacterial plasmids. In: *Encyclopedia of Life Sciences*. John Wiley and Sons. https://doi.org/10.1002/9780470015902.a0029193

9. Tolmasky ME, Alonso JC (2015) *Plasmids: Biology and Impact in Biotechnology and Discovery*. Wiley.

